# Work related etiology of de Quervain’s tenosynovitis: a case-control study with prospectively collected data

**DOI:** 10.1186/s12891-015-0579-1

**Published:** 2015-05-28

**Authors:** Stéphane Stahl, Daniel Vida, Christoph Meisner, Adelana Santos Stahl, Hans-Eberhard Schaller, Manuel Held

**Affiliations:** Department of Plastic, Hand and Reconstructive Surgery, Burn Center, BG-Trauma Center, Eberhard-Karl University, Schnarrenbergstr. 95, 72076 Tübingen, Germany; Eberhard-Karl University of Tübingen, Institute for Clinical Epidemiology and Applied Biometry, Silcherstr. 5, 72076 Tübingen, Germany; Department for Plastic Surgery, Marienhospital Stuttgart, Böheimstr. 37, 70199 Stuttgart, Germany

**Keywords:** de Quervain stenosing tenosynovitis, de Quervain’s tenosynovitis, Etiopathogenesis, Occupational disease, Manual labor

## Abstract

**Background:**

The etiology of de Quervain's tenosynovitis (dQ) has been based on conflicting small case series and cohort studies lacking methodological rigor. A prospective case-control study was conducted to analyze the most common risk factors for dQ.

**Methods:**

Between January 2003 and May 2011, 189 patients surgically treated for dQ vs. 198 patients with wrist ganglia (WG) (controls) were identified in our clinic’s electronic database. Sample characteristics, exertional, anatomical, and medical risk factors were compared between groups.

**Results:**

dQ vs. WG differed by average age (52 vs. 43 years) and gender ratio (15/62 vs. 26/39). No significant difference between dQ vs. WG was found after subgrouping professional activities (manual labor: 18 % vs. 26 %, respectively, *p* = 0.23). No asymmetric distribution of comorbidities, wrist trauma, forceful or repetitive manual work, or medication was observed.

**Conclusions:**

Neither heavy manual labor nor trauma could be shown to be predisposing risk factors for dQ.

## Background

On average, approximately 0.5 % of men and 1.3 % of women of working age suffer from de Quervain's tenosynovitis (dQ) [1], resulting in two million working days lost per year in Germany [2]. dQ is believed to result from repetitive, forceful, and ergonomically stressful work, from anatomic variations, hormonal influences or pregnancy, rheumatoid disease, trauma, or drugs, such as fluoroquinolone. A systematic review and meta-analysis regarding the etiology of dQ has revealed that expert opinions and case reports account for almost 60 % of the relevant literature [3]. The meta-analysis performed to evaluate the strength of the association between dQ and physical exposure included five contradicting cohort studies which lacked methodological rigor regarding the diagnosis of dQ, the classification of physical exposures, and the consideration and control of bias and confounding.

Research on the etiology of dQ is of prime importance for the following reasons: 1) in an analogy to antirheumatic treatment, treating the underlying causality of dQ may reduce the need for surgical treatment; 2) evidence of causality is a prerequisite for the recognition of dQ as an occupational disease by The International Labour Organization (ILO), the World Health Organization (WHO) and European Union (EU) [4–6]; 3) effective prevention mandates the avoidance of causal factors which would lower the incidence or likelihood of progression of dQ; and 4) basic research is discouraged by the general acceptance of traditional hypotheses in textbooks without questioning the underlying evidence.

An objective and reliable diagnosis of dQ is a precondition to the scientific investigation into its etiology. However, the diagnosis of dQ is often based on the interpretation of patients’ self-reporting of symptoms and inconsistent pain-provoking tests. In a prospective clinical study of 104 patients, the specificity of the Finkelstein test performed by two experienced hand surgeons was estimated to average 0.14 after complementary X-ray and ultrasonography examinations (sensitivity: 0.89) [7]. The numerous differential diagnoses of dQ, the examiner-dependent variability in performing pain-provoking tests, variations in pain perception and expression, and secondary gain in an occupational setting may further hinder a reliable diagnosis of dQ. Symptom relief after treatment has, therefore, been considered the gold standard of diagnosis [8].

This paper investigates the association of dQ and the most frequently discussed risk factors in a case-control study. We hypothesized that overuse, anatomic variation, rheumatoid disease, trauma, or fluoroquinolone medication would be associated with dQ.

## Methods

The study was conducted with the approval of the Ethics Review Board of Eberhard-Karls-University, Tuebingen, Germany (approval number 466/2011BO2).

A total of 189 consecutive patients with dQ and 198 consecutive patients with wrist ganglia (WG) (controls) treated between January 2003 and May 2011 were identified for this case-control study. Patients younger than 18 years of age were excluded. Patients with wrist ganglia were chosen as a control group because these patients were free of the outcome of interest, were representative of the general population, and because different etiopathologies are associated with dQ vs. wrist ganglia. In particular, patients with wrist ganglia display similar demographics, including age and handedness, when compared with the rest of the population [9].

The presence of wrist ganglia was confirmed during surgery. The diagnosis of dQ was confirmed by post-operative pain relief. None of the patients presented both pathologies. Pre-operative diagnosis of dQ was made by a board certified hand surgeon. Examination included the precise location of tenderness, palpation of abnormal tendon gliding, a clinical test (as described by Eichhoff), and standardized PA and lateral X-ray examinations of the wrist [10]. All patients who provided their written informed consent and who completed the case report form (including a medical record evaluation, a self-assessment questionnaire, and an interview and a clinical examination) were included in the study.

### Patient's electronic and paper-based medical record evaluation

Data were collected retrospectively from medical records and prospectively from a case report form during an interview and clinical examination. Data retrieved from the standardized medical records included the dates of examinations, any preceding trauma or surgery, medication at the time of initial diagnosis, and hand X-rays suggestive of arthritis. All operative reports were screened for the presence of an accessory compartment and the presence of two or more tendons of the extensor pollicis brevis (EPB) or of the abductor pollicis longus (APL).

### Self-assessment questionnaire

The development of the self-assessment questionnaire, containing demographic, occupational, and medical items, has been previously described [11]. Questions regarding demographic data assessment were placed at the end of the questionnaire to minimize respondent fatigue bias. The questionnaire contained the following item categories:

1. Demographic data: age, sex, ethnicity, height, weight and handedness.
2. Occupational data: a detailed description of job characteristics and long-term exposure to stressful hand positioning during work-related, computer, sport, and musical activities was obtained. The highest educational degree achieved, the highest secondary education achieved, and the current work-related situation was also assessed. The current primary occupation, a detailed description of the main task of this occupation, the duration, and the average number of working hours per week were also assessed, along with the frequency of twisting, powerful motions, unusual hand positions, lifting, pushing or pulling of heavy loads, and ergonomic aspects of computer use. The last part of the questionnaire concerned the patient’s former occupation and contained the same questions regarding job characteristics and long-term exposure to stressful hand-positioning as the section regarding current occupation.
3. Leisure activities: a detailed description of sport and musical activities and computer use at leisure was requested. In addition to information about type and duration of sports practiced and of musical instruments played on a regular basis, the time spent using a computer and the ergonomics of keyboard and computer-mouse were considered.
4. Medical data: questions assessed the clinical symptoms of rheumatoid arthritis as defined by the American College of Rheumatology, 1987 (morning stiffness lasting at least 1 h, symmetric joint pain, pain of 3 or more large joints, painful hand joints [12], and symptoms of Raynaud syndrome [13]), as well as symptoms of carpal tunnel syndrome, trigger digits and basal thumb arthritis [14]. Smoking habits and alcoholic consumption were also assessed.

### Interview and clinical examination

The standardized clinical examination assessed previous injuries of the upper extremity, their characteristics (fractures, contusions, lacerations) and dates prior to the diagnosis of dQ, as well as associated surgeries. Employment data (including years of active employment, occupational skill level, and job category), duration of work incapacity, prior surgery, medication before and at the time of diagnosis, range of motion and grip strength [15], tenderness, and signs of accompanying diseases of the hand (rheumatoid nodules and radiographic signs of joint degeneration, trigger digit, carpal tunnel syndrome) were also included in the standardized clinical examination. Carpal tunnel syndrome was confirmed by nerve conduction studies and electromyography upon reporting of nocturnal pain or paresthesia and a positive Durkan test. The average intensity of manual work was assessed on an ordinal scale and the duration of exertion was estimated in hours per shift using a modified validated questionnaire in a face-to-face interview [16].

**Fig. 1:**
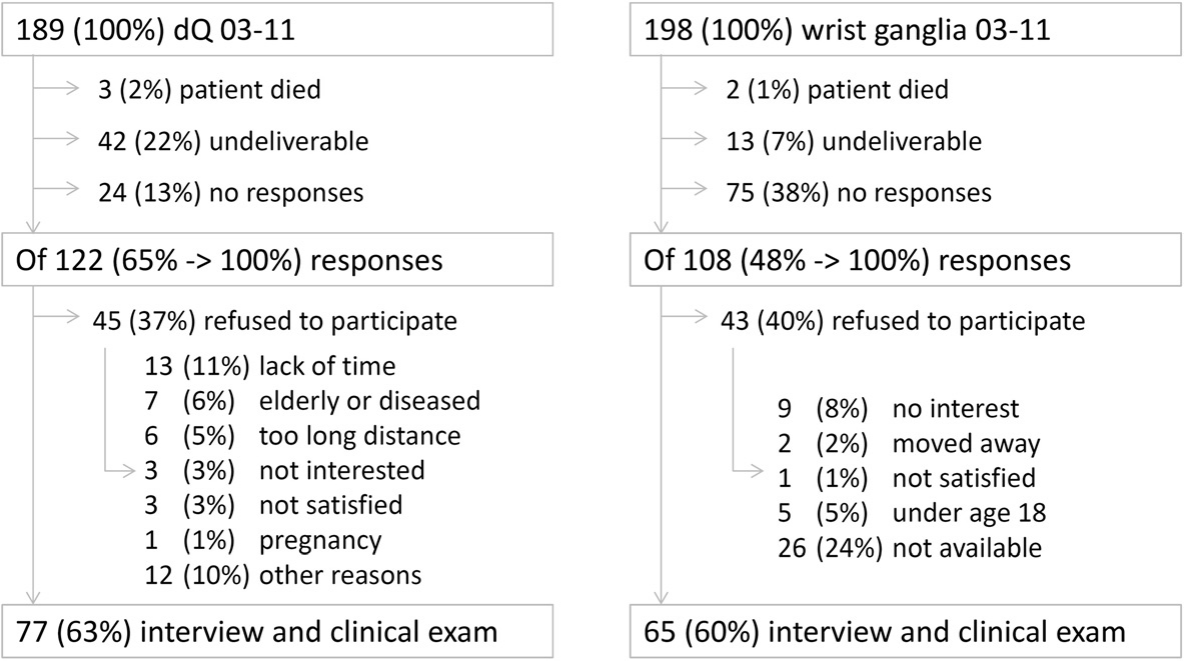
Flow diagram demonstrating the individual steps of this study

The self-assessment questionnaire was delivered by mail in November 2012 to 387 patients and was re-sent 6 weeks later to non-responders. If no response was received in the following 6 weeks, the patients were contacted by telephone, and an appointment for the clinical examination was arranged, when the questionnaire was then given to the patient (Fig. 1).

The self-assessment questionnaire assessed risk-exposures during the week preceding the completion of the examination to minimize recall bias. The responses were only evaluated if the respondent affirmed that the week referred to was representative of his/her normal work week. However, to prevent indication bias, the repetitiveness, force, and posture of the current and previous employment was assessed during the interview. The occupational profiles of patients who changed their job and those who continued performing the same job since the diagnosis of dQ were compared to test for the healthy worker effect. To control for recall bias, the only working conditions evaluated were those in which the patients had been performing the same job since the diagnosis of dQ.

Data were retrieved from the paper-based and electronic patient records to compensate for missing data [17]. Inconsistencies were resolved during the interview. The accuracy and completeness of the case report form was verified after the clinical examination to avoid missing data and to eliminate any misunderstanding. To minimize further response fatigue, characteristics of risk exposure (such as the duration and frequency of applied force during typical daily tasks) were assessed during the interview. Participants were offered transportation/compensation of costs for a hospital visit.

### Statistical analysis

Fisher exact test and chi squared test were used to compare categorical variables between cases and controls. The unadjusted odds ratios and the associated 95 % confidence intervals (CIs) were estimated using the Mantel-Haenszel-method. An unconditional multivariate logistic-regression model was planned to estimate odds ratios and the associated 95 % CIs for risk factors of dQ. This model was planned only if there are variables which had remarkable unadjusted odds ratios (lower limit of the CI odds ratio >1.0) and if the prevalence of the risk factor was > 10 % in both groups. T-Tests for independent groups were used to compare age and BMI between cases and controls. Mann Whitney *U* Test was used to compare the mean working hours per week between cases and controls. A p value <0.05 for associations was considered statistically significant without adjustments for multiple testing. SAS 9.2 (SAS Institute Inc. Cary, NC), and SPSS 20 (IBM Corp, Released 2012, IBM SPSS Statistics for Windows, Version 20.0, Armonk. NY: IBM Corp) were used for all analyses.

## Results

Of the 189 referred patients with confirmed dQ diagnosis (compared with 198 controls), 65 % (122/189) responded to the postal and telephone inquiries (compared with 54 % for controls). Of these 122 patients with dQ (compared with 108 controls), 77 agreed to participate (compared with 65 controls), while 45 could not be contacted or refused to participate (compared with 43 controls). Seventy-seven patients with dQ (compared with 65 controls) were examined between November 2012 and February 2013 and were included in this study (Fig. 1). Age distribution of the control group (average: 43 years; median: 42 years) was representative of the general German population (average: 45 years; median: 43 years) [18]. Patients with dQ were significantly older than the controls (Table 1, p < 0.001). There was a strong female predominance among dQ patients (81 % females with dQ vs. 51 % females in the general German population) [18].

**Table 1:** Subject demographics

Characteristics	Cases	Controls	Unadjusted OR (95 % CI)	p-value
	n ( %)	n ( %)		
Male gender	15/77 (19)	26/65 (40)	2.7 (1.2–6.3)	0.01
Mean age (yrs) [median; range]	52 [53; 18–82]	43 [42; 18–78]	.	<0.001
Age > 50 years	49/77 (64)	19/65 (29)	0.2 (0.1–0.5)	<0.001
Caucasian ethnicity	77/77 (100)	62/65 (95)	n.a.	0.1
Height (cm) [median; range]	168 [166; 156–187]	171 [172; 155–204]	-	
BMI [median; range]	26 [26; 19–35]	26 [26; 19–35]	-	0.45
Right handedness	69/76 (91)	58/65 (89)	1.2 (0.3–4.2)	0.78

There was no difference between the dQ cases and controls with regards to handedness or smoking. Thirty-four patients with dQ vs. 13 patients with WG had been unemployed for 12 months or had never been employed (*p* = 0.01) (data not shown). Two patients (5 %) changed their job after receiving a diagnosis of dQ (compared with four controls (7 %); *p* = 1.0). However, the occupational profiles were not significantly different between patients who changed their occupational activity after the diagnosis of dQ vs. those who did not (data not shown).

### Repetitive, forceful, and ergonomically stressful work and leisure activities

dQ patients and controls did not differ significantly regarding the type of work (manual labor vs administrative work; *p* = 0.23; Table 2) and professional groups (*p* = 0.43). No asymmetric distribution of professional hierarchy was observed between the two groups according to Goldthorp [19] (*p* = 0.95).

**Table 2:** Etiopathological factors in order of decreasing frequency

Variables	Cases n ( %)	Controls n ( %)	OR (95 % CI)^k^	p - value
Professional groups	n = 58^e^	n = 52^e^		0.43^a^
semi-skilled worker	21/58 (36)	12/52 (23)		
training occupation	22/58 (38)	27/52 (52)		
technical occupation	6/58 (10)	5/52 (10)		
academic profession	9/58 (16)	8/52 (15)		
Occupational group	n = 77^d^	n = 65^d^		0.23^a^
manual labor	14/77 (18)	17/65 (26)		
administrative work	34/77 (44)	20/65 (31)		
neither manual labor nor administrative work	29/77 (38)	28/65 (43)		
Occupational hierarchy according to Goldthorp	n = 58^e^	n = 52^e^		
I	13/58 (22)	9/52 (17)	I – IV vs. V – VII	0.95^a^
			0.7 (0.3 – 1.6)	
II	13/58 (22)	11/52 (21)		
III	9/58 (16)	7/52 (13)		
IV	6/58 (10)	4/52 (8)		
V	5/58 (9)	6/52 (12)		
VI	6/58 (10)	7/52 (13)		
VII	6/58 (10)	8/52 (15)		
Working time	n = 58^e^	n = 52^e^		
average working hours per week, in hours (range)	34 (7–60)	38 (10–55)		0.05^c^
Piecework	n = 58^f^	n = 52^f^		
daily	4/58 (7)	3/52 (6)	Yes vs. never	0.76^b^
			1.6 (0.5 – 5.5)	
3/4 of the time	1/58 (2)	-		
1/2 of the time	1/58 (2)	3/52 (6)		
1/4 of the time	2/58 (3)	1/52 (2)		
never	50/58 (86)	45/52 (87)		
Work with computer	n = 58^f^	n = 47^f^		
daily	17/58 (29)	15/47 (32)	Yes vs. never	0.99^b^
			1.5 (0.6 – 3.7)	
3/4 of the time	2/58 (3)	2/47 (4)		
1/2 of the time	4/58 (7)	2/47 (4)		
1/4 of the time	12/58 (21)	9/47 (19)		
never	23/58 (40)	19/47 (40)		
Twisting motions at work	n = 58^f^	n = 52^f^		
every hour	11/58 (19)	11/52 (21)	Every hour/daily vs. 2–3× per week/	0.31^b^
			1× per week/never	
			1.2 (0.6 – 2.8)	
daily	14/58 (24)	15/52 (29)		
2–3× per week	5/58 (9)	-		
1× per week	4/58 (7)	3/52 (6)		
never	24/58 (41)	23/52 (44)		
Powerful motions of the forearm or hand	n = 58^f^	n = 47^f^		
every hour	14/58 (24)	8/47 (17)	Every hour/daily vs. 2–3× per week/	0.06^b^
			1× per week/never	
			1.0 (0.4 – 2.3)	
daily	10/58 (17)	15/47 (32)		
2–3× per week	4/58 (7)	2/47 (4)		
1× per week	2/58 (3)	7/47 (15)		
never	28/58 (48)	15/47 (32)		
Pulling or pushing of loads	n = 58^f^	n = 47^f^		
every hour	3/58 (5)	5/47 (11)	Every hour/daily vs. 2–3× per week/	0.04^b^
			1× per week/never	
			0.6 (0.3 – 1.5)	
daily	8/58 (14)	12/47 (26)		
2–3× per week	3/58 (5)	-		
1× per week	11/58 (19)	2/47 (4)		
never	33/58 (57)	28/47 (60)		
Ergonomically stressful work	n = 58^f^	n = 52^f^		
every hour	7/58 (12)	9/52 (17)	Every hour/daily vs. 2–3× per week/	0.42^a^
			1× per week/never	
			0.7 (0.3 – 1.5)	
daily	10/58 (17)	15/52 (29)		
2–3× per week	4/58 (7)	3/52 (6)		
1× per week	6/58 (10)	6/52 (12)		
never	31/58 (53)	19/52 (37)		
Musical instrument	n = 77^h^	n = 65^h^		
current	4/77 (5)	7/65 (11)	0.6 (0.1–2.3)	0.35^b^
former & never	73/77 (95)	58/65 (89)		
High impact sports ^i^	n = 77^h^	n = 65^h^		
current	39/77 (51)	23/65 (35)	1.8 (0.9–3.7)	0.06^b^
former & never	38/77 (49)	42/65 (65)		
Rheumatoid arthritis				
suspicion of rheumatoid arthritis ^j^	0/77 (0)	1/65 (2)	-	0.46^b^
morning stiffness > 1 h	4/77 (5)	14/65 (20)	0.8 (0.1–5.1)	0.01^b^
arthritis of 3 or more joint areas	3/77 (4)	7/65 (11)	0.4 (0.1–1.6)	0.2^b^
symmetric arthritis	0/77 (0)	3/65 (4)	-	0.1^b^
Trauma (<2 years before dQ)	2/77 (3)	0/65 (0)	-	0.5^b^
Working time at computer	n = 28^h^	n = 30^h^		
Computer use in hours per week	19 (1–40)	16 (1–54)		0.24^c^
Keyboard	n = 28^h^	n = 30^h^		
ergonomic	2/28 (7)	1/30 (3)	Laptop vs. PC (normal/ergonomic)	0.88^b^
			0.9 (0.2 – 6.6)	
normal	23/28 (82)	25/30 (83)		
Laptop	3/28 (11)	4/30 (13)		
Mouse	n = 28^h^	n = 30^h^		
normal	24/28 (86)	26/30 (87)	Touchpad vs. PC-mouse (normal/ergonomic)	0.87^b^
			0.9 (0.1 – 6.4)	
ergonomic	2/28 (7)	1/30 (3)		
Touchpad	2/28 (7)	3/30 (10)		

Two patients with dQ and four controls who changed their job after diagnosis were not taken into account in the characterization of professional activity. The professional training of all patients was evaluated, regardless of whether they were working at the time of the diagnosis or not

^a^Chi2-Tests

^b^Fisher’s exact test

^c^Mann-Whitney *u* test

^d^Total number of patients in whom the item is applicable

^e^Total number of patients working at the time of diagnosis

^f^Total number of patients in whom the professional activity includes this kind of work

^g^Number of patients, which are doing or have done this kind of activity

^h^Restricted to sports that require repetitive and strenuous forearm and wrist movements

^i^ Patients with at least 3 of the symptoms below

^j^Adjusted for age (≤50 vs. >50) and gender

No association with manual force levels ≥ “high” as defined by Steinberg [16] was found in either group (powerful motions: *p* = 0.06, pulling or pushing of loads: *p* = 0.04). No difference was observed regarding repetitive work (piecework, *p* = 0.76), twisting motions (*p* = 0.31), or inconvenient working positions (*p* = 0.42) (Table 2). Length of computer use, type of keyboard of mouse used did not differ between dQ cases vs. controls (Table 2).

The frequency of leisure activities, such as high impact sports (as defined by Bancroft [20]) and the frequency of practice and the type of musical instrument performed did not differ between the two groups (high impact sports: *p* = 0.06; use of a musical instrument: *p* = 0.35) (Table 2).

### Anatomic variations

Of the 77 patients included in the study, an accessory compartment was observed in 34 % and the presence of two or more tendons of the EPB and the APL was noted in 7 % and 12 %, respectively (Table 3).

**Table 3:** Studies referring to the anatomic variations within the first dorsal compartment of the wrist including the results presented herein

	First author and year of publication	Male gender	Accessory compartment ( %)	≥2 EPB ( %)	≥2 APL ( %)
Patients with dQ	Mc Dermott et al. (2012)	6/40	21/40		
	Choi et al. (2011)	0/13 (15 wrists)	11/15	1/15	11/15
	Kwon et al. (2010)	8/40 (43 wrists)	19/43	NM	NM
	Gousheh et al. (2009)	7/50	43/50	NM	NM
	El-Hadidy et al. (2006)	17/62	20/62	28/62	39/62
	Minamikawa et al. (1991)	32/70	33/70	2/70	66/70
	Own results	15/77	26/77	5/77	9/77
Anatomic studies	Shiraishi et al. (2005)	41/80 (159 wrists)	49/159	21/159	156/159
	Gonzalez et al. (1995)	-/66	31/66	0/66	57/66
	Minamikawa et al. (1991)	-/71	53/71	NM	NM
	Leslie et al. (1990)	-/50 (100 wrists)	34/100	NM	NM
	Leao et al. (1958)	20/27 (50 wrists)	13/50	2/50	37/50
	First author and year of publication	Male gender	Accessory compartment ( %)	≥2 EPB ( %)	≥2 APL ( %)
Patients with dQ	Mc Dermott et al. (2012) [39]	6/40	21/40 (53)		
	Choi et al. (2011) [51]	0/13 (15 wrists)	11/15 (7)	1/15 (7)	11/15 (73)
	Kwon et al. (2010) [52]	8/40 (43 wrists)	19/43 (44)	NM	NM
	Gousheh et al. (2009) [30]	7/50	43/50 (86)	NM	NM
	El-Hadidy et al. (2006) [32]	17/62	20/62 (32)	28/62 (45)	39/62 (63)
	Minamikawa et al. (1991) [23]	32/70	33/70 (47)	2/70 (3)	66/70 (94)
	Own results	15/77	26/77 (34)	5/77 (6)	9/77 (12)
Anatomic studies	Shiraishi et al. (2005) [33]	41/80 (159 wrists)	49/159 (31)	21/159 (13)	156/159 (98)
	Gonzalez et al. (1995) [31]	-/66	31/66 (47)	0/66 (0)	57/66 (86)
	Minamikawa et al. (1991) [23]	-/71	53/71 (75)	NM	NM
	Leslie et al. (1990) [53]	-/50 (100 wrists)	34/100 (34)	NM	NM
	Leao et al. (1958) [29]	20/27 (50 wrists)	13/50 (26)	2/50 (4)	37/50 (74)

NM, not mentioned

### Comorbidities

Application of the ACR criteria for rheumatoid arthritis, 1987 resulted in three cases suspicious for rheumatoid arthritis in dQ cases (compared with one control; Table 2). Carpal tunnel syndrome was significantly associated with dQ (dQ: 12/77; controls 2/65; *p* = 0.01; data not shown). Trigger digits were also more frequent among dQ patients (dQ: 10/77; controls: 3/65; *p* = 0.14; data not shown).

### Trauma

Two patients with confirmed dQ diagnoses (compared with 0 controls; *p* = 0.5) reported trauma before the onset of symptoms. One patient suffered a distal radius fracture after a fall and a contusion of the wrist from a falling tree branch while gardening at 8 months and 10 months, respectively, before the diagnosis of dQ (Table 2).

### Drug-induced

None of the patients included in this case-control study reported receiving fluoroquinolone medication at the time of diagnosis.

## Discussion

To our knowledge, this is the first case-control study to analyze the association between dQ and its frequently discussed etiopathological factors (repetitive, forceful and ergonomically stressful work and leisure activities, anatomic variations, rheumatoid disease, trauma, and fluoroquinolone medication), including one of the largest series of dQ patients reported in literature [21–23].

### Repetitive, forceful, and ergonomically stressful work and leisure activities

Unlike previous studies who made no distinction regarding physical exposure, a qualitative and quantitative assessment of physical exposure was undertaken to determine an association between repetitive, forceful, and ergonomically stressful work and dQ [24–26]. Four cross-sectional studies assessed 585 people who had professional activities suspected of triggering dQ. A total of 34 cases of dQ were found (6 %) [25–28]. Repetitive, forceful and ergonomically stressful hand motions at work were assessed in only one study which did not find a significant association [22]. In the present study, the unemployment rate was similar in both groups after adjusting for age and gender. The strong female predominance among dQ patients compares well with previous studies [22].

### Anatomic variations

Our findings were similar to previous reports of dQ patients regarding the number of EPB and APL tendons and the presence of an accessory fibrous septum subdividing the first extensor compartment. In two retrospective case series of 50 dQ patients each, an accessory compartment was found in 26 % and 86 %, respectively [29, 30]. Anatomical and clinical studies have reported two or more EPB tendons present in 0 – 45 % of cases [31, 32]. Twenty-four percent of dQ patients had two or more APL tendons, while previous studies reported an incidence of 63 – 98 % [32, 33] (Table 3).

Several anatomic studies and one clinical study have reported supernumerary APL or EPB tendons and an accessory compartment as suspected causes of increased gliding resistance in dQ patients resulting in an inflammatory response [34–36]. However, anatomical studies and case series did not find a significant difference in the number of tendons of dQ patients vs. cadavers [23, 37, 38]. Two other retrospective case series did not find a significantly higher number of APL and EPB tendons among dQ patients [23, 39]. Anatomical variations are a stable condition after embryological development. If anatomical variations in the number of tendons or compartments were the cause of dQ, the possible reasons for the delay between skeletal maturity and the occurrence of dQ at an average age of 52 years must be explained.

### Trauma

One expert opinion and one case report reported trauma as the suspected cause of dQ based solely on the temporal sequence of trauma followed by the diagnosis of dQ [40, 41]. To the best of our knowledge, there are no studies showing a significant association between trauma and the development of dQ or experimental studies which suggest that trauma is a causal factor.

### Drug-induced

We have identified three case reports of elderly patients who developed tendinopathy (achilles tendinitis) after fluoroquinolone medication [42–44]. Two experimental studies in rodents have found toxic effects of various fluoroquinolones on the Achilles tendon sheath [45, 46]. Similar effects have been discussed as the cause of tendinopathy within the first dorsal compartment of the wrist [42, 47, 48].

### Strengths and weaknesses of the study

The information regarding potential risk factors relied on self-reporting (a potential source of confirmation bias, recall bias, respondent fatigue bias, and interviewer bias). However, to minimize respondent fatigue bias and interviewer bias, the occupational risk factors were assessed both in the self-assessment questionnaire and in the standardized interview. Because this case-control study was performed as a singlecenter study in a workers’ compensation clinic, a recruitment bias may have caused the overestimation of potential occupational risk factors. Therefore the control group was chosen from the same hospital to control for recruitment bias. Because the study focuses on work-related risk factors, hormonal risk factors such as pregnancies were not examined. There have been no previous reports of common etiological factors between dQ and WG. However, the association of dQ with pregnancies and the older age of patients with dQ suggest different etiopathologies.

## Conclusions

In conclusion, dQ does not appear to relate to exertion of thumb musculature or anatomical variation. Caucasian women aged ≥ 60 years have a higher risk of developing dQ. Like dQ, primary osteoarthritis is age and sex related and more prevalent in Europe. However, the susceptibility genes of osteoarthritis have been also been associated to tendon pathologies [49]. This suggests that susceptibility genes of dQ may be discernible from a molecular perspective, requiring future research [50].

## Abbreviations

dQ: Quervain's tenosynovitis
WG: Wrist ganglia
